# Congenital disorders of glycosylation (CDG): state of the art in 2022

**DOI:** 10.1186/s13023-023-02879-z

**Published:** 2023-10-19

**Authors:** Rita Francisco, Sandra Brasil, Joana Poejo, Jaak Jaeken, Carlota Pascoal, Paula A. Videira, Vanessa dos Reis Ferreira

**Affiliations:** 1 CDG & Allies - Professionals and Patient Associations International Network (CDG & Allies-PPAIN), Department of Life Sciences, School of Science and Technology, Universidade NOVA de Lisboa, 2819-516 Caparica, Portugal; 2 UCIBIO - Applied Molecular Biosciences Unit, Department of Life Sciences, NOVA School of Science and Technology, Universidade NOVA de Lisboa, 2819-516 Caparica, Portugal; 3 Associate Laboratory i4HB, Institute for Health and Bioeconomy, NOVA School of Science and Technology, Universidade NOVA de Lisboa, 2819-516 Caparica, Portugal; 4 Center for Metabolic Diseases, Department of Pediatrics, KU Leuven, 3000 Louvain, Belgium; 5 Portuguese Association for Congenital Disorders of Glycosylation (CDG), Department of Life Sciences, NOVA School of Science and Technology, Universidade NOVA de Lisboa, 2819-516 Caparica, Portugal

**Keywords:** Congenital disorders of glycosylation (CDG), Rare diseases, Disease classification, Nosology

## Abstract

Congenital disorders of glycosylation (CDG) are a complex and heterogeneous family of rare metabolic diseases. With a clinical history that dates back over 40 years, it was the recent multi-omics advances that mainly contributed to the fast-paced and encouraging developments in the field. However, much remains to be understood, with targeted therapies' discovery and approval being the most urgent unmet need. In this paper, we present the 2022 state of the art of CDG, including glycosylation pathways, phenotypes, genotypes, inheritance patterns, biomarkers, disease models, and treatments. In light of our current knowledge, it is not always clear whether a specific disease should be classified as a CDG. This can create ambiguity among professionals leading to confusion and misguidance, consequently affecting the patients and their families. This review aims to provide the CDG community with a comprehensive overview of the recent progress made in this field.

**Supplementary Information:**

The online version contains supplementary material available at 10.1186/s13023-023-02879-z.

## Introduction

Congenital disorders of glycosylation (CDG) are a peculiar group of inherited metabolic diseases (IMD). Contrary to other IMD families, they are due to defects occurring in several cell organelles, mainly the cytosol, the endoplasmic reticulum (ER), the ER-Golgi intermediate compartment, the Golgi, and the sarcolemmal membrane [1]. The defects are associated with glycoprotein and glycolipid glycan assembly and remodeling. Since glycans are essential for the function of these proteins and lipids, defects within glycosylation pathways can usually impact multiple organs and cause various symptoms that can manifest from birth [2]. The most typical CDG symptoms are associated with neurological and developmental disabilities [3]. Still, their multisystem nature also causes serious hepatic, gastrointestinal, and hormonal problems that require close and continuous healthcare [4].

The high variety of CDG clinical manifestations and biological pathways has led to difficulties in defining a clear and universal classification and nomenclature for this group of disorders. The first attempt at classifying CDG dates back to 1999 [5], and was based on the serum transferrin isoelectrofocusing (IEF) pattern (e.g., CDG-Ia). In 2008, as the number of reported CDG exponentially increased, the first alphabetically and chronological CDG system was replaced by a novel nomenclature system comprising the name of the gene of the individual CDG diagnosis (e.g. PMM2-CDG) and maintained until today [6, 7]. Nevertheless, it is not always clear whether a metabolic disorder should be classified as a CDG because a number of CDG have several features in common with other metabolic diseases [8]. In 2022 it was proposed to create an international advisory group of experts in the field of CDG to discuss and determine whether a disorder should be classified or not as a CDG [9].

So far, 163 known CDG genetic defects encompass 193 different phenotypes. The heterogeneity of CDG is striking from several points of view. The large majority (~ 88%) are multisystem diseases [10]. The mono-system diseases (~ 12%) affect either the brain, eyes, skin, skeleton, skeletal muscles, liver, red blood cells, or neutrophils [10–12]. Even though all are rare, for some CDG only single digit numbers of patients have been reported, while at the other end of the spectrum, there is PMM2-CDG with more than one thousand patients diagnosed over 40 years. The severity of clinical expression extends from perinatal death (and probably even miscarriage) to mild adult involvement [13]. The heterogeneity is even more pronounced since a gene defect can result in multiple clinical presentations depending on the involved variant. For example, EXT2-CDG is associated either with the mono-organ disorder exostoses type 2 (MIM: 133701), affecting only the skeleton, or with a multisystem syndrome (MIM: 616882) characterized by dysmorphia, seizures, scoliosis, and macrocephaly [14]. The same is true for POFUT1-CDG, leading to either a skin disorder (MIM: 615327) or a multisystem disorder encompassing microcephaly and global developmental delay with cardiac and vascular features [15].

CDG genetic transmission is usually autosomal recessive (AR). Seven percent of the clinical presentations have an autosomal dominant (AD) transmission, and 6% are X-linked (XL). Epigenetic defect has been reported only in XYLT1-CDG. This phenotypic and genetic heterogeneity hampers CDG diagnosis except in the minority of patients with a recognizable phenotype (e.g., exostoses in EXT1/EXT2-CDG) [10, 16].

Treatment is nearly exclusively symptomatic since a more or less efficient and established basic treatment (with mannose) is only available for MPI-CDG, a CDG limited to the liver and the intestine. Nevertheless, in the last years, research has led to the discovery of novel biomarkers and disease models. Currently, there are four ongoing observational studies (NCT04201067, NCT02089789, NCT04198987, and NCT03404856), including two natural history studies (NCT03173300 and NCT01417533) and four therapeutic clinical trials (NCT04833322, NCT04679389, NCT03404869, and NCT03404856) [17]. The fact that most CDG involve the brain constitutes a significant barrier to treatment [18].

This paper presents a comprehensive and structured overview of all CDG identified until the end of 2022, and discusses glycosylation pathways, phenotypes, genotypes, inheritance patterns, biomarkers, disease models, treatments, and dates of first reports of the different phenotypes. The main goal of this mini-review is to update the CDG community on the progress made over the last years.

## Materials and methods

For this review, we used a combination of specific keywords related to the different CDG [e.g., the gene names individually or conjugated with CDG; clinical signs and symptoms; disease models (mouse, drosophila, yeast, zebrafish) and biomarkers] to search the Medline database, using PubMed as the search engine [19]. The OMIM database [20] was used to extract the information relative to the human genotype–phenotype and their characteristics, whereas the Uniprot database [21] was consulted to collect information related to the protein function and biochemical pathway. For each CDG recent papers were privileged, particularly those reviewing the literature and describing large patient cohorts. The selected articles were read and the ones matching the selection criteria were included.

Inclusion criteria comprised:

- Only English-written manuscripts;
- Articles reporting biomarkers, in vitro and in vivo models, clinical signs, and symptoms;
- Recently published reviews.

The exclusion criteria were the following:

- Knockdown in vitro models (cellular-based), knock-in transient cell-based models, and disease models exploring the role of glycogenes for other diseases (e.g., in cancer).
- Models that do not recapitulate a human phenotype.

An advisory committee composed of four CDG professional experts and one CDG family member provided expert guidance during article selection and throughout manuscript development.

## Results

The primary objective of this concise review is to provide the CDG community with an update on the advancements achieved in recent years. All the information, gathered until the end of 2022, is summarized in the Additional file 1: Table S1 and is discussed in the next paragraphs.

**Fig. 1:**
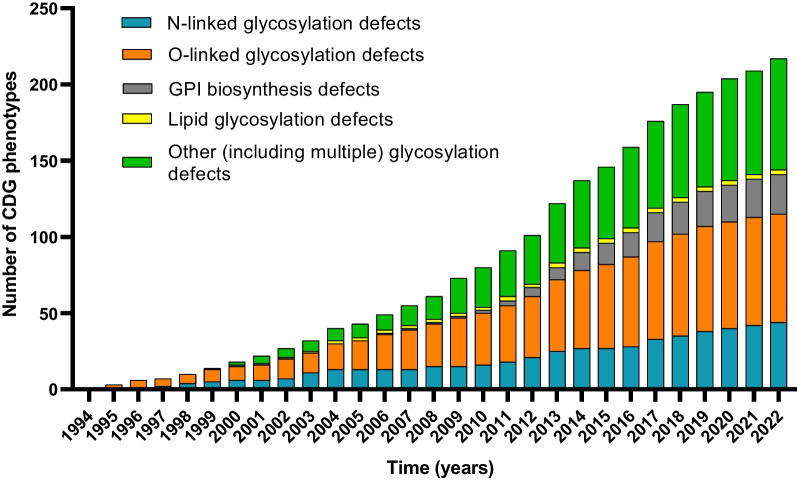
Graphical representation of the yearly distribution of the newly reported CDG phenotypes (1994–2022) according to the underlying affected glycosylation pathway(s). The years correspond to when the association between the gene and the phenotype was established

Over the years, CDG have been classified according to the affected glycosylation pathways, namely N-glycosylation, O-glycosylation, glycosylphosphatidylinositol (GPI)-anchor synthesis, lipid glycosylation, and other (including multiple) glycosylation pathways (Fig. 1). The latter category includes defects impairing vesicular transport (e.g., COG defects), activated sugar transport (e.g., SLC35C1-CDG, MIM: 266265), monosaccharide synthesis and interconversion (e.g., FCSK-CDG, MIM: 618324) and V-ATPase pumps (e.g., ATP6AP2-CDG, MIM: 301045), among others. The subcellular location of the defect can also be used as a complementary CDG classification criterion, e.g., N-linked defects are mostly limited to the ER, and O-linked defects are mainly located in the Golgi [22].

**Fig. 2:**
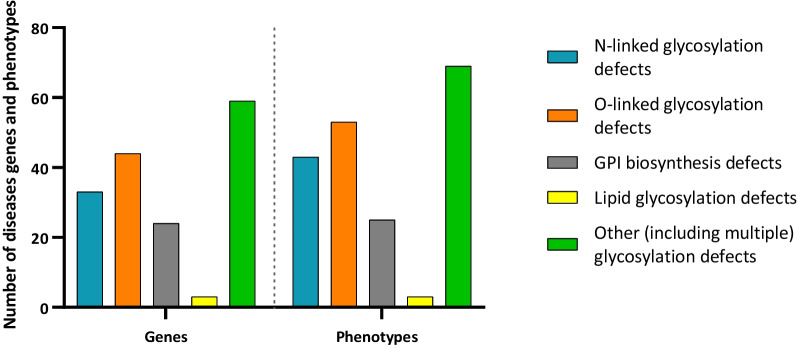
Total number of genes and phenotypes related to defects in each glycosylation pathway, namely N-linked glycosylation, O-linked glycosylation, GPI biosynthesis, lipid glycosylation, and other (including multiple) glycosylation defects

To date, 163 genes have been associated with 193 disease phenotypes linked to CDG (Fig. 2). N-linked glycosylation disorders (n = 43) are caused by variants in 33 genes; the 53 O-linked glycosylation disorders are caused by defects in 44 genes; GPI biosynthesis defects (n = 25) are due to variants affecting 24 genes, while variants in 3 genes cause the 3 lipid glycosylation defects. The 69 disorders affecting other (including multiple) glycosylation pathways described are caused by defects in 59 genes (Fig. 2).

The majority of CDG have an AR inheritance pattern (n = 161) (Table 1). However, other inheritance patterns have been described. Autosomal dominant patterns have been described in N-linked (n = 6), O-linked (n = 5), and other (including multiple) glycosylation pathway defects (n = 4). X-linked defects (n = 12), both of dominant (XLD) and recessive (XLR) inheritance, have been described in all glycosylation pathways except for lipid glycosylation defects. Examples include SLC37A4-CDG (AD, MIM: 602672), ALG13-CDG (XL, MIM: 300884), and ATP6AP1-CDG (XLR, MIM: 300972). Of note, XLD inheritance (n = 1) has only been reported in other (including multiple) glycosylation pathway defects (i.e., SLC35A2-CDG, MIM: 300896). Additionally, de novo variants have been reported in CDG, mainly among ALG13-CDG females, Saul-Wilson syndrome and SLC35A2-CDG [23–25].

**Table 1:** CDG inheritance patterns per glycosylation defects

Glycosylation defects	Inheritance patterns					
	AR	AD	XL	XLR	XLD	NA
N-linked	30	6	1	3		3
O-linked	45	5	0	2		1
GPI	24	0	0	1		0
Lipid	2	0	0	0		1
Other (including multiple)	60	4		4	1	

*AR* Autosomal recessive, *AD* Autosomal dominant, *XL* X-linked, *XLR* X-linked recessive, *XLD* X-linked dominant, *NA* Information not available

Due to this molecular variety, CDG display high intra- and inter-disease clinical heterogeneity. Moreover, as is true for other genetic diseases, intrafamiliar variability has always to be kept in mind. Variants in the same gene presenting different inheritance patterns have been linked to different CDG phenotypes. This is the case of EXT2, whose AR inherited disease variants lead to seizures, scoliosis, and macrocephaly syndrome (MIM: 616682), while AD variants cause the multiple exostoses phenotype (MIM: 133701). Different variant types [e.g., loss and gain-of-function (GOF) variants] have also been associated with particular diseases, namely COG4- (MIM: 618150) and GNE- (MIM: 269921) CDG. Furthermore, the variant type and location within the gene can affect phenotypic severity, with more severe phenotypes usually being associated with greater disruption of the involved enzyme, transporter or chaperone. This has been documented for B3GALT6-CDG (Al-Gazali syndrome, MIM: 609465), and CANT1-CDG (Desbuquois dysplasia, MIM: 251450), among others [26–28]. Specific variants and genotypes have also been linked to particular CDG phenotypes. Examples are the PIGL p.L176P variant that, in compound heterozygosity, causes colobomas, congenital heart defects, migratory ichthyosiform dermatosis, intellectual disability, and ear anomalies (MIM: 280000) and the GORS2 p.V144L variant which produces progressive myoclonic epilepsy 6 (MIM: 614018). CDG phenotypic diversity and severity can be influenced by other determinants. Reported modifiers include additional defective glycogenes and mitotic intragenic recombination [29, 30].

Most CDG are complex clinical conditions, affecting practically all organs and thus leading to a large number of different symptoms/syndromes [22] as dystroglycanopathies, cardiomyopathy, skeletal dysplasia, cutis laxa, Ehlers-Danlos syndrome, congenital myasthenia syndromes a.o.. A few mono-organ or pauci-organ CDG have been reported, such as DHDDS-CDG (MIM: 613861), with one phenotype only associated with a form of familial retinitis pigmentosa, GNE-CDG (MIM: 605820) that manifests as a progressive myopathy and GANAB-CDG presenting as a polycystic kidney or liver diseases (MIM: 600666).

**Fig. 3:**
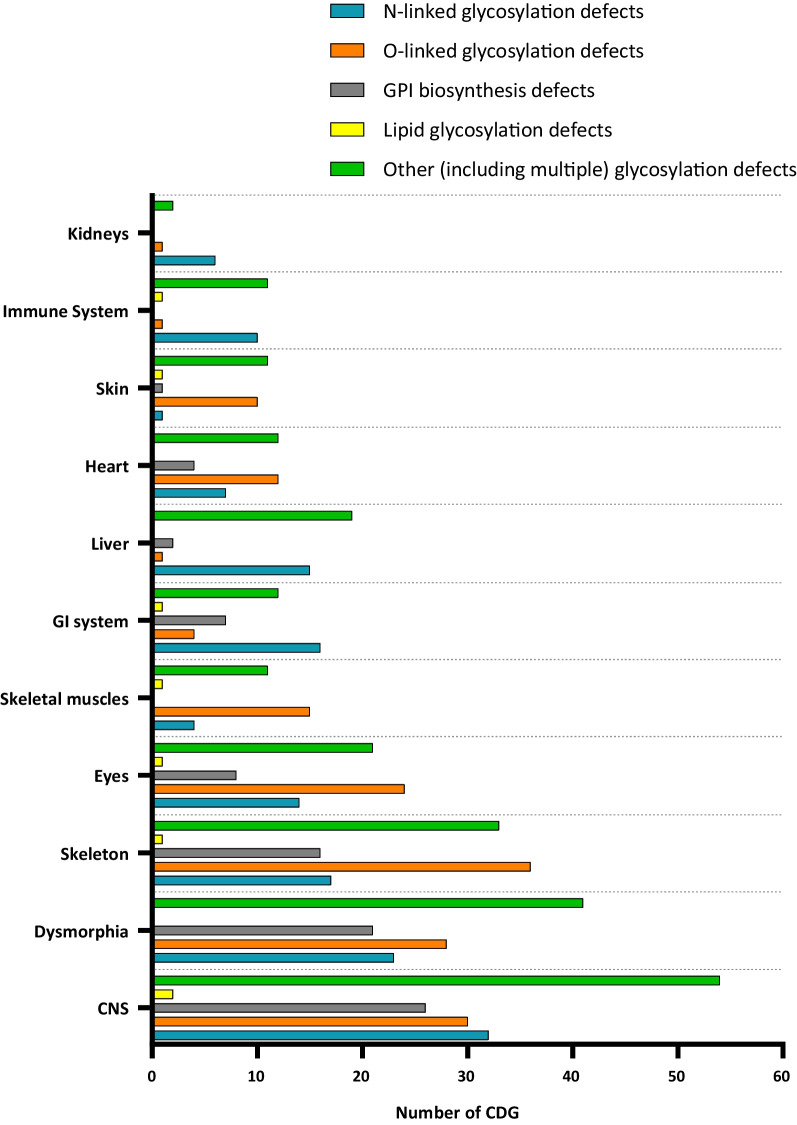
Organs involved in the different CDG groups. Legend: CNS—central nervous system; GI—gastrointestinal

The most affected system across the majority of CDG is the central nervous system (CNS; n = 144) (Fig. 3). Common neurological signs and symptoms include intellectual disability, hypotonia, cerebellar ataxia, nystagmus, seizures, dysarthria, and dysphagia. Besides neurologic involvement, most CDG patients present with variable dysfunction of other organs and systems, like dysmorphism (n = 113), and failure to thrive (Fig. 3). After the CNS, the skeleton (n = 103) is the most commonly affected organ in all CDG groups, except for lipid glycosylation defects. The skeletal muscle (n = 15) and the eyes (n = 24) are commonly affected organs among O-linked glycosylation defects. Among the other (including multiple) glycosylation pathway defects, the eyes (n = 21) and the liver (n = 19) are the most affected systems (Fig. 3). For both N-linked glycosylation and GPI biosynthesis defects, the skeleton, the GI system, and the eyes are the most frequently involved (Fig. 3).

In the last years, several biomarkers have been developed, increasing the chances to identify and discover novel CDG. One example is the two-dimensional electrophoresis of haptoglobin β glycoforms [31], which has been helpful as a complementary biomarker to identify novel CDG like SLC37A4-CDG (MIM: 602672) and SLC10A7-CDG (MIM: 618363).The analysis of bikunin, by western blotting, allowed the identification of some linkeropathies [32] like B3GAT3-CDG (MIM: 245600), B4GALT7-CDG (MIM: 130070), B3GALT6-CDG (MIM: 615349 and 271640), as well as the identification of some other (including multiple) glycosylation pathways defects such as ATP6V0A2-CDG (MIM: 219200), CCDC115-CGD (MIM: 616828), COG5-CDG (MIM: 613612) and COG7-CDG (MIM: 608779). In addition, a more sensitive and specific method was developed using flow injection-electrospray ionization-quadrupole time-of-flight mass spectrometry (ESIQTOF-MS) for serum N-glycan profiling [33], allowing the identification of novel characteristics of polymannose changes in CDG like DDOST-CDG (MIM: 614507), SSR4-CDG (MIM: 300934) and ALG3-CDG (MIM: 601110). Whole Exome Sequencing and Whole Genome Sequencing techniques have become very powerful tools to diagnose CDG. For CDG without known biomarkers, it will be the only way to diagnose a patient. Examples are defects in *B4GALNT1, FCSK, GFUS, GNPNAT1, PIGG, POFUT1* (skin presentation)*, POGLUT1* (skin presentation)*, SLC35D1, ST3GAL3 and TGDS.*

Despite the discovery of several biomarkers and the development of several disease models for CDG, very few targeted therapies exist for this group of disorders, and most available therapies are restricted to symptom management. In fact, guidelines for the clinical management of CDG are only available for MPI-, PMM2-, and PGM1-CDG [13, 34, 35]. Currently, effective and targeted therapeutics available are mannose supplementation and liver transplantation for MPI-CDG (MIM: 602579) [34], heart transplantation for DOLK-CDG (MIM: 610768) and galactose supplementation for PGM1-CDG (MIM: 614921) [18, 35, 36]. To overcome the lack of targeted therapies for CDG, several treatment strategies are being investigated, mainly dietary supplementation with sugars and trace elements (e.g., vitamin), which is is the case of dietary sugar supplementation with fucose (e.g., FUT8-, GFUS- and SCL35C1-CDG) or galactose supplementation (e.g., TMEM165-, SLC39A8-, SLC35A2-, PGM1-, ALG13- and PMM2-CDG), most being administered under compassionate and off-label use programs.

Additional therapeutic avenues under investigation are drug repurposing, and gene replacement strategies [18, 37, 38]. One example of drug repurposing is the open-label, single-patient compassionate study on PMM2-CDG with epalrestat, an aldose reductase inhibitor used for treating diabetic neuropathy [39, 40]. Despite all the research being developed for CDG therapies, until 2022, most of these treatments have not been approved by regulatory bodies or are available in the market [31].

## Discussion

Disease classification can be a complex process. It can suffer from shortcomings such as the lack of a clear disease-causing mechanism or widespread input from the stakeholders involved (researchers, clinicians, patients, and their families) [8]. The first CDG classification system (sub)classified the N-glycosylation defects alphabetically (e.g., CDG-Ia, CDG-IIa, etc.) and was based on the serum transferrin pattern obtained by IEF, the gold standard screening technique for N-glycosylation defects with sialic acid deficiency [6, 7]. However, new research studies have unveiled new CDG pathophysiological mechanisms leading to the description of new disease phenotypes and to the reclassification of already-known disorders as CDG [1].Well-known examples of the latter are the muscular dystrophy-dystroglycanopathies. Since the first biochemical and genetic characterization of PMM2-CDG in 1995 and 1997, respectively, the number of described CDG has increased exponentially [41]. The development of new techniques for CDG diagnosis, namely lipid-linked oligosaccharides by HPLC, glycan analysis by mass spectrometry, and whole exome/genome sequencing, has contributed to an exponentially increased detection of variants in more than 160 genetic *loci* for CDG. For example, in the last five years, deficiencies have been identified in seven GPI synthesis genes, namely GPAA1-, PIGB-, PIGH-, PIGK-, PIGP-, PIGS-, and PIGU-CDG. In the same period, 12 N-linked and 13 multiple glycosylation pathway defects were described. A few examples of N-linked glycosylation defects include ALG10-CDG, ALG14-CDG (MIM: 616227 and 619036), EDEM3-CDG, MAGT1-CDG (MIM: 301031), and more recently MAN2A2-CDG. Furthermore, variants in the X-linked MAGT1 causing hypoglycosylation led to the re-classification of MAGT1 deficiency as a CDG, which was previously only associated with a primary immunodeficiency with a magnesium transport defect (XMEN) [42]. A novel pathogenic variant causing a combined immune deficiency, abnormal glycosylation, and lysosomal involvement was described as MAN2B2-CDG. However, patients present with normal transferrin isoelectric focusing profiles, and only mild glycosylation changes were observed by ESI-QTOF in the blood [43]. Some examples of other (including multiple) glycosylation pathway defects discovered in the last five years are ATP6VI1-, GO7- (Congenital myasthenic syndrome), GET4-, GFUS- and GNPNAT1-CDG, and most recently CAMLG-CDG. Novel variants were also identified in *CSGALNACT1* and *EXTL3*, causing CSGALNACT1-CDG and EXTL3-CDG, two new disorders affecting the O-linked glycosylation pathway, particularly the glycosaminoglycan (GAGs) biosynthesis [44, 45].

Molecularly, glycosylation is “the synthesis of fully functional glycans and their covalent enzymatic attachment to other molecules including proteins, lipids, and small RNA” [39]. However, these functions are performed in the presence of various enzymes, donor and acceptor substrates, metal ions, and depend also on an adequate pH. Hence, CDG are caused by inborn pathogenic (recessive, dominant, or X-linked) or de novo variants in the genes encoding proteins involved in the different glycosylation steps but also genes affecting closely linked and essential steps in glycosylation as mentioned above [9]. In addition, different phenotypes can be caused by different inheritance patterns. This is the case for ALG8- and ALG9-CDG, in which AR variants are responsible for a different phenotype than the one caused by AD variants. Another cause for differences in the phenotypic presentation linked to inheritance patterns is mosaicism. This is observed for SLC35A2-CDG, which has a dominant X-linked inheritance pattern and in which the only identified affected males were somatic mosaics. The lack of non-mosaic-affected males suggests that a wild-type *SLC35A2* allele is required for survival. Nearly all CDG are monogenic disorders caused by pathogenic alleles transmitted by a mendelian, monogenic inheritance pattern. This means that most CDG are caused by variants affecting only one gene. Some disorders have also been reclassified as CDG as the expansion of their pathophysiological mechanisms has included underlying glycosylation defects (e.g., Saul-Wilson syndrome [*COG4*], Cowden syndrome 7 [*SEC23B*], ALG5- and ALG9-CDG) [41].

Concluding, this paper refers to published data/knowledge as it stands at 2022. There is a steadily increasing number of reported CDG. It is not always straightforward if a specific disease should be classified as CDG. This can create confusion and misguidance, impacting professionals, patients and their families. During the 2021 World CDG Conference and the 2021 Scientific CDG Symposium, the importance of defining a precise CDG nomenclature and nosology was discussed. This highlights the essential need to capture the complementary expertise from the CDG community (researchers, health professionals, patients, and caregivers) to discuss and define the criteria to include or exclude a disease as a CDG.

### Supplementary Information

**Additional file 1. **CDG state of the art until 2022: glycosylation pathways, phenotypes, genotypes, inheritance patterns, biomarkers, disease models, and treatments.

## Data Availability

Not applicable.

## Abbreviations

AD: Autosomal dominant
AR: Autosomal recessive
CDG: Congenital disorders of glycosylation
CNS: Central nervous system
ER: Endoplasmic reticulum
ESIQTOF-MS: Injection-electrospray ionization-quadrupole time-of-flight mass spectrometry
GI: Gastrointestinal
GPI: Glycosylphosphatidylinositol
IEF: Isoelectrofocusing
IMD: Inherited metabolic diseases
XL: X-linked
XLD: X-linked dominant
XLR: X-linked recessive
